# Foreign DNA detection by high-throughput sequencing to regulate genome-edited agricultural products

**DOI:** 10.1038/s41598-020-61949-5

**Published:** 2020-03-18

**Authors:** Takeshi Itoh, Ritsuko Onuki, Mai Tsuda, Masao Oshima, Masaki Endo, Hiroaki Sakai, Tsuyoshi Tanaka, Ryo Ohsawa, Yutaka Tabei

**Affiliations:** 1 Bioinformatics Team, Advanced Analysis Center, National Agriculture and Food Research Organization, Tsukuba, Ibaraki 305-8602 Japan; 2 National Institute of Agrobiological Sciences, Tsukuba, Ibaraki 305-8602 Japan; 3 Tsukuba Plant Innovation Research Center, University of Tsukuba, Tsukuba, Ibaraki 305-8572 Japan; 4 Institute of Agrobiological Sciences, National Agriculture and Food Research Organization, Tsukuba, Ibaraki 305-8634 Japan; 5 Institute of Crop Science, National Agriculture and Food Research Organization, Tsukuba, Ibaraki 305-8518 Japan; 6 Present Address: Research Institute, National Cancer Center Japan, Chuo-ku, Tokyo 104-0045 Japan

**Keywords:** Biotechnology, Computational biology and bioinformatics

## Abstract

Although the advent of several new breeding techniques (NBTs) is revolutionizing agricultural production processes, technical information necessary for their regulation is yet to be provided. Here, we show that high-throughput DNA sequencing is effective for the detection of unintended remaining foreign DNA segments in genome-edited rice. A simple *k*-mer detection method is presented and validated through a series of computer simulations and real data analyses. The data show that a short foreign DNA segment of 20 nucleotides can be detected and the probability that the segment is overlooked is 10^−3^ or less if the average sequencing depth is 30 or more, while the number of false hits is less than 1 on average. This method was applied to real sequencing data, and the presence and absence of an external DNA segment were successfully proven. Additionally, our in-depth analyses also identified some weaknesses in current DNA sequencing technologies. Hence, for a rigorous safety assessment, the combination of *k*-mer detection and another method, such as Southern blot assay, is recommended. The results presented in this study will lay the foundation for the regulation of NBT products, where foreign DNA is utilized during their generation.

## Introduction

Several current biotechnology techniques, such as genome editing, have been developed to produce novel crops and livestock in an effective manner^1–3^. While their regulation has been initiated, they are currently not overseen in some areas^4–7^, and their status is, in many cases, still under debate, especially if foreign DNA sequences are used in the technologies and subsequently removed from the final product^8–10^. Because current high-throughput sequencing technologies are promising for the detection of foreign DNA remnants in host genomes^11–16^, guidelines for large-scale sequence data submission have been developed for the safety assessment of genetically-modified organisms (GMOs)^17,18^. However, although PCR-based methods have been widely used for the validation of genome-edited agricultural products (GEAPs)^19–21^, unlike GMOs, the number of reports focused on massive sequencing-based methods for GEAPs is still relatively limited^22,23^. A study focused on the statistical aspect of detecting unauthorized DNA materials in GMOs was previously reported^24^, but a general theoretical framework for data analysis of foreign DNA-free GEAPs has yet to be established. Here, using rice as a model crop genome, we conducted a large-scale bioinformatics study of both simulated and real data and present fundamental information that will be necessary for regulations for new breeding techniques (NBTs). In particular, as low-cost DNA decoding of the sequencing-by-synthesis method (SBS) is anticipated to play a pivotal role for the detection of unintended remaining foreign DNA segments, sequence data generated by the HiSeq platform are the focus in this study.

Strictly speaking, even a single base pair insertion of an exogenous nucleotide can create a GMO (e.g., legislation of the Cartagena act of the Ministry of the Environment, Japan). It is also noteworthy that, since such a short stretch of DNA is indistinguishable from other intrinsic regions, it could be excluded from consideration for the purpose of practical assessment, as it would not entail a risk of health damage. It was previously shown that a DNA stretch of 20 nucleotides (nt) in length can be unique in an organism, such as in humans, and would be distinguishable^1,8^. This calculation was based on the simple assumption that a DNA sequence is randomized; however, real biological sequences are more or less biased such that real data should show different patterns. In this study, to propose a lower boundary for the DNA length to be detected for practical regulations, we count the number of *k*-mer patterns (nucleotide segments of length *k*), which have been used for a wide variety of studies^25,26^, and examine their uniqueness as real biological sequences.

Although the importance of next-generation sequencing (NGS) for the GEAP/GMO safety assessment is recognized, the results of NGS data analyses vary depending on the bioinformatics analytical programs used^27^, and this could hamper the standardization of regulation process for GEAPs and related materials. In fact, guidelines from the European Commission for sequencing data submission require detailed descriptions about programs and their usage^17^. Moreover, for a general short-read mapping and SNP calling procedure, a high-quality reference genome is needed, and this raises a serious problem. First, a genuine high-quality reference genome sequence is not available for most crops except rice. Second, the rice genome is still incomplete^28^, and in this way, it will not be possible to obtain a 100% completely deciphered genome. Additionally, an individual crop for which NBT is applied may have a genome that is slightly different from its reference due to spontaneous mutations. For this reason, in a strict sense, the complete genome of the individual has to be determined if a comparison with a reference is required. However, as long as only the unintended remnants of foreign DNA used during the NBT process are under consideration, one can focus on the detection of the foreign DNA, such as a vector, in a data set generated by a high-throughput sequencer. Here, we present a simple method to unambiguously detect unintended remaining foreign DNA segments that compares *k*-mers between the genome and vector sequences without a reference genome sequence. This method was validated through computer simulation and using real data analysis of the rice genome, which currently has the best quality among the genomes of agricultural organisms^28^. The method was also applied to the bread wheat genome^29^, which is largest among the currently available complete crop genomes. The limitations and weaknesses of the *k*-mer analysis and the amount of DNA sequence data needed for this analysis were also investigated.

## Results

### Foreign DNA detection by *k*-mer analysis

There are several popular computer programs to align short SBS reads to a genome. Although this type of read-mapping is a standard way to process NGS data, different programs may produce different results, so it should be difficult to standardize the bioinformatics analysis for the regulation of GEAPs. To examine the inconsistency of short-read mapping programs, 50× of simulated coverage data incorporating 0.3% artificial errors in a random manner were generated from the rice genome, and these artificial reads were mapped to the genome itself by the following three programs: BWA-MEM, NovoAlign and SOAPaligner. The numbers of mapped read pairs varied significantly among the programs (Supplementary Table 1), as these programs employ different algorithms to reduce the search time, and therefore the results are not necessarily same. However, in the case of the search for all identical DNA sequences, their results should be unambiguously the same at all times.

**Figure 1:**
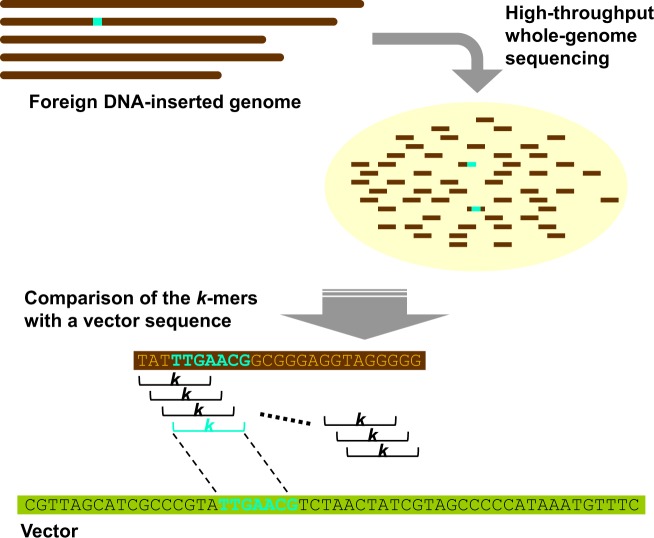
Overview of the *k*-mer detection analysis. A whole genome sample, which possibly contains a specific amount of unintended foreign DNA, is experimentally fragmented and sequenced. All the *k*-mers for each read are compared with the vector sequence used and the identical hits (light blue regions and nucleotides) are recorded.

**Figure 2:**
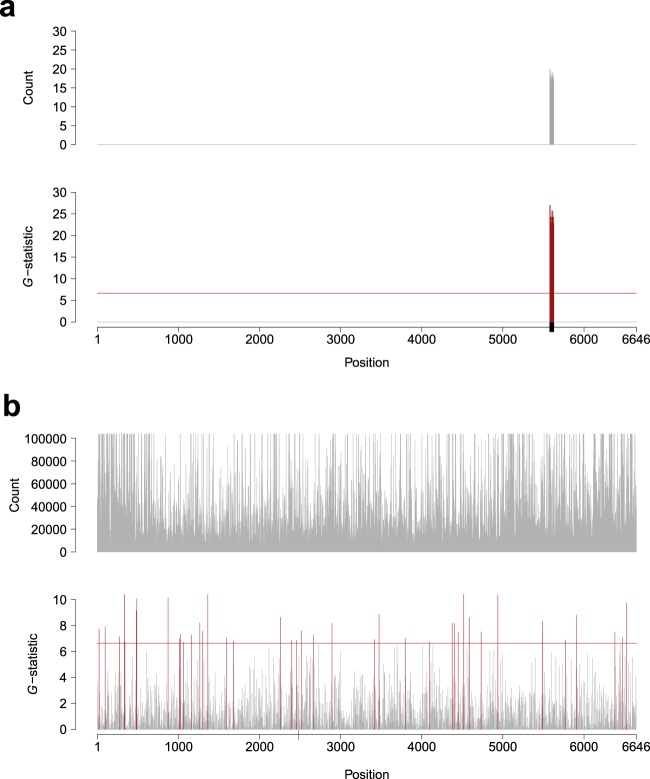
Detection of identical *k*-mers between the rice genome and vector sequences by computer simulation. (**a**) A 100-nt insert and 50-mers, and (**b**) a 10-nt insert and 10-mers. The y-axes are for the count and *G*-statistic of a *k*-mer at each nucleotide position. The x-axis indicates the nucleotide positions on a vector sequence. The position where a foreign DNA segment was computationally inserted was depicted by a black box on the x-axis: 5,577–5,676 in (**a**) and 2,483–2,492 in (**b**). The red horizontal line corresponds to the 1% significance level (*G* = 6.634) and the bars exceeding this line are also drawn in red. The number of 10-mer counts over 100,000 and *G*-statistics over 10 are omitted in (**b**). For the complete version, see Supplementary Fig. 1.

To find unintended remaining foreign DNA in a host genome, we employed a short identical DNA segment detection method in which *k*-mers were extracted from all the reads and were compared with the vector sequence (Fig. 1). A series of computer simulations were conducted to examine the validity of this method (Fig. 2). First, a 100-nt segment clipped from the ColE1 plasmid sequence that was used as a model for the cloning vector in this study was inserted *in silico* into the rice genome, and paired-end reads with 50× coverage were computationally generated from this simulated genome. The 50-mers for all the reads derived from the rice genome were compared with the ColE1 sequence and the number of *k*-mer occurrences at each nucleotide position was counted. As a result, the inserted 100-nt region was perfectly detected with no false hits (Fig. 2a, Supplementary Table 2). Second, if a 10-nt insertion was examined by 10-mers (Fig. 2b and Supplementary Fig. 1), the true signal was virtually invisible among the false hits because the same patterns of extremely short DNA sequences, such as the 10-nt sequence, naturally occur in the rice genome as well. In fact, the *G*-statistic of the true 10-mer fell below the significance level and all the significant hits were erroneous (Supplementary Table 2). These results indicate that there should be a technical and statistical limitation for short DNA segment detection by means of large-scale sequencing. In the subsequent analyses, we aimed to examine simulated and real data sets so as to lay a foundation for sequencing-based regulations for NBT.

### Shared identical *k*-mer patterns between species

The lower detection limit for the short foreign DNA segments depends primarily on the genome size, and 20 nt was thus advocated as an appropriate limit for genomes of the size of maize or human^1,8^. If a DNA segment of length *k* is considered, the theoretical total number of all possible nucleotide patterns is 4^*k*^, and hence the expected occurrence frequency of a specific 20-mer in a randomized nucleotide sequence of 1 trillion bp is less than 1. It is obvious that, if *k* is small enough, a genome should contain all the patterns. Here, we examined the real genome sequence data of five species and one plasmid and found that the large genomes of some species, such as rice and wheat, encompass all the patterns if *k* is 10 (Supplementary Figs. 2 and 3, Supplementary Table 3). Since the number of *k*-mer patterns does not exceed the genome size and there should be redundant patterns, the theoretical upper limit of this number is no more than the total number of nucleotides in the genome. As *k* increases, the total number of patterns grows exponentially, and the genomes have quite a small portion of *k*-mer patterns (Supplementary Figs. 2 and 3, Supplementary Table 3). If *k* is 20, the rice genome contains merely 0.049% of the patterns, and even the gigantic wheat genome, which is over 10 Gbp^29^, harbors as few as 0.533% of the patterns (Supplementary Table 3).

As biological sequences are certainly biased, if two genome sequences are compared, the number of identical *k*-mer patterns may differ from the random expectation. Here, we compared the rice genome sequence and the complete sequence of the ColE1 plasmid^28,30^. Using a *k* of 15, the host species genome contains thousands of vector nucleotide patterns, which is more than the expectation, and there is still a possibility that 20-nt segments can be shared between the host and the vector (Table 1). The four 20-mers shared between rice and ColE1 were derived from the complementary strands of two genomic positions and show no specific conserved features among the species, such as ribosomal DNA regions (Supplementary Fig. 4). In the case where such an extremely short segment is integrated into a genome, they may not be distinguishable from the intrinsic DNA. However, if the DNA segment length is 25 or more, there is a subtle possibility that they are identical to any DNA sequence portion from the host, as we see no identical 25-nt sequences between rice and ColE1 (Table 1).

**Table 1:** *K*-mer patterns shared between rice and ColE1.

*k*	Number of the *k*-mer patterns shared between rice and ColE1	
	Expected*	Observed
15	3,947	5,378
20	6	4
25	0	0

*Note: The expected numbers are based on the real numbers of corresponding *k*-mers in rice and ColE1.

### Validation of the *k*-mer method by computer simulation

If short DNA sequences of 20–30 nt in length are unique between species, it might be required to detect these short segments through regulated processes. To examine the limitations of short identical DNA segment detection by means of massive sequencing, we have conducted the following large-scale in-depth computer simulation. DNA segments of 15, 20, 30 and 50 nt in length were randomly cut out from the ColE1 sequence and were computationally inserted into the rice genome in a random manner. The detection of true hits for several *k*-mers was investigated 1,000 times (Table 2). As a result, the 10-mer analysis did not find the true signals and the 15-mer analysis failed several times to identify the 20- and 30-nt inserts. It is notable that when a 20-mer or more was examined, no failure was observed. Another concern of this *k*-mer-based detection is the number of false positives, which should be minimized during the practical assessment process. As the ColE1 sequence is 6,646 bp, it was expected that approximately 66 hits would be observed from randomly generated sequences at the 1% significance level. In our data, for 10-mer, more than 70 false hits were detected on average (Table 3). This is understandable because the nucleotide patterns of the 10-mers are indistinguishable between the rice genome and the ColE1 sequence (Fig. 2b, Supplementary Figs. 2 and 3). Although the 15-mer analysis still showed a high false-positive ratio, the 20-mer and more were even less error-prone and there was less than 1 false hit on average (Table 3). These results indicate that if high-throughput DNA sequencing is conducted in an ideal manner, an unintended 20-nt foreign DNA insert could be captured in the rice genome with a quite low false positive ratio.

**Table 2:** Number of successes over 1,000 iterations in the detection of foreign DNA segments.

Insert length	10-mer	15-mer	20-mer	25-mer	30-mer	35-mer	40-mer	45-mer	50-mer
15 nt	88	1000	—	—	—	—	—	—	—
20 nt	123	987	1000	—	—	—	—	—	—
30 nt	217	999	1000	1000	1000	—	—	—	—
50 nt	395	1000	1000	1000	1000	1000	1000	1000	1000

**Table 3:** Average number and standard deviation of false positive hits in the detection of foreign DNA segments.

Insert length	10-mer	15-mer	20-mer	25-mer	30-mer	35-mer	40-mer	45-mer	50-mer
15 nt	70.66 ± 8.86	32.52 ± 6.27	—	—	—	—	—	—	—
20 nt	70.38 ± 8.79	32.64 ± 6.21	0.69 ± 0.96	—	—	—	—	—	—
30 nt	70.13 ± 8.39	32.52 ± 6.03	0.73 ± 1.01	0.70 ± 0.98	0.69 ± 0.98	—	—	—	—
50 nt	70.27 ± 8.49	32.68 ± 6.35	0.83 ± 1.55	0.80 ± 1.37	0.78 ± 1.23	0.77 ± 1.12	0.76 ± 1.05	0.75 ± 1.02	0.75 ± 1.02

Throughout the aforementioned simulations, we used 50× coverage sequences, which is more than the 40× coverage required previously for GMO detection^17^, though the amount of sequencing data necessary for our analysis of GEAPs was unclear. Here, we conducted another computer simulation in which the average sequencing depth was changed from 10× to 50× in the rice genome and the ratios of the cases where the 20-nt foreign DNA insert was successfully detected by 20-mer analysis were examined for each coverage through 1,000 iterations (Table 4). It is clear that 10× was too shallow to detect a short-segment insert of foreign DNA. In the case of 20×, one of the 1,000 iterations failed to detect the simulated 20-nt insert. There was no failure if 30× or more reads were used; thus, it is expected that the possibility that the true signal is overlooked is less than 10^−3^. Additionally, the numbers of false positive hits were quite small in all cases (Supplementary Table 4). Hence, it would be recommended that the average sequencing depth be at least 30× after low-quality read regions are trimmed by an appropriate program. Furthermore, there is another concern that even though the sequencing depth appears to be sufficient, there may remain a specific portion of unsequenced genomic regions. If the nucleotides to be sequenced are selected in a completely random manner and the sequencing coverage is over 100, the probability that any one of the nucleotides is non-selected is less than 10^−43^ under the assumption of a Poisson distribution, so a genome of a regular length is expected to be fully sequenced. However, the process of DNA sequencing is nonrandom because DNA fragmentation, library construction, etc. may incorporate various types of bias. In fact, the 140.1× coverage data set from a wild type rice sample was mapped to the rice genome by BWA-MEM and 59,411 (0.0159% of the genome) unsequenced/undetected nucleotides were found (Supplementary Table 5). The 157.7× coverage data for another independent sample also had 58,207 (0.0156%) unsequenced/undetected nucleotides when the reads were mapped by BWA-MEM, and two other programs showed much worse mapping results (Supplementary Table 5). These results indicate that regardless of reasons, we cannot completely exclude the possibility that a number of nucleotides might remain unexamined even though deep sequencing is conducted.

**Table 4:** Detection accuracy of a 20-nt insert by 20-mer analysis depending on the coverage.

**Coverage**				
10x	20x	30x	40x	50x
78.0%	99.9%	100.0%	100.0%	100.0%

### Validation of the *k*-mer method via real data analysis

**Figure 3:**
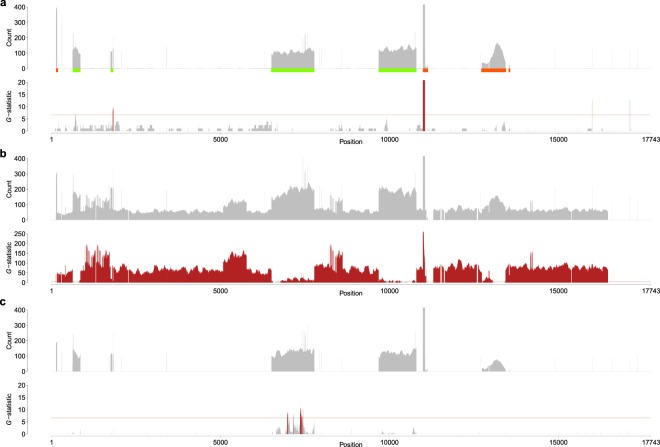
Detection of identical 20-mers between the real genome and vector sequences. For details, see the legend of Fig. 2. Data obtained from the (**a**) wild type, (**b**) T_0_ and (**c**) T_1_ samples are shown. The regions that were derived from rice (green boxes) and were identical to contaminated DNA (orange boxes) are depicted in the panel (**a**). The number of 10-mer counts over 400 and *G*-statistics over 20 (**a**,**c**) or 250 (**b**) are omitted, and the complete version is drawn in Supplementary Fig. 6.

As an application example of the *k*-mer detection method, the genome-edited rice generated by CRISPR/Cas9 was validated by the 20-mer detection analysis. The genomic DNA from four samples (two wild types, one T_0_ line and one T_1_ line) were sequenced by the Illumina HiSeq X platform. After trimming of the Illumina reads by Trimmomatic (See Supplementary Note 1), 52,292,878,177 nt (195,606,982 read pairs), 58,863,113,535 nt (219,755,791 read pairs), 60,730,824,843 nt (219,200,039 read pairs) and 54,313,317,595 nt (198,414,540 read pairs) were obtained from the two wild types, the T_0_ line and the T_1_ line, respectively. Prior to DNA sequencing, the vector-free nature of the T_1_ sample was confirmed by a PCR experiment (Supplementary Fig. 5); thus, no vector-like sequences were expected to remain in either the wild type or T_1_ samples. Nonetheless, the 20-mer analysis of the wild type sample found that several consecutive 20-mers were shared between rice and the vector (Fig. 3a and Supplementary Fig. 6). First, the regions indicated by green boxes in the vector were derived from the rice genome. Hence, it is naturally understandable that large numbers of 20-mer counts are observed in those regions; even if a statistically significant excess of 20-mers are observed in the wild type, they are regarded as false hits detected purely by chance. Second, the two 20-nt regions at the 16,002–16,021 and 17,122–17,141 nt positions happened to be identical to specific rice genomic sequences and probably were detected because of random fluctuations in the data. Last, it turned out that 20-mer hits in the regions indicated with orange boxes in Fig. 3a originated from a contamination in the sequencing library kit used in this study and its details are described in Supplementary Note 2 and Supplementary Figs. 7 and 8.

In comparison with the wild type, the DNA sequencing data extracted from the genome-edited T_0_ generation sample clearly showed significant signals in a vector-wide manner (Fig. 3b and Supplementary Fig. 6), which is consistent with a Southern blot assay (Supplementary Fig. 5a). In contrast, the T_1_ generation sample had some signals within a rice-derived region only (Fig. 3c and Supplementary Fig. 6). If the cisgenic regions are excluded from this analysis, there were no significant hits in the T_1_ sample, and this result is consistent with our PCR experiment (Supplementary Fig. 5b). Additionally, the 50-mers were analyzed for the same data set and essentially the same results were obtained with a more clear-cut picture (Supplementary Fig. 9).

To further validate the applicability of the *k*-mer detection method, a sequencing data set obtained from the genome-edited wheat genome was examined^31^. As a result, we observed results similar to those of rice, in which a T_0_ sample showed significant signals and there were essentially no hits in a T_1_ sample that was expected to be a null segregant (Supplementary Figs. 10 and 11). In this way, the unintended integrated foreign DNA segments can be detected easily by searching for identical *k*-mers, and the absence of this foreign DNA can also be confirmed in both rice and wheat without profound bioinformatics knowledge.

## Discussion

In this study, we presented a number of basic and essential data necessary for the regulation of foreign-DNA-free GEAPs. Whilst the clinical application of genome-editing should require a thorough genome-wide investigation of off-target mutations^32^, in the case of agricultural products, the focus should be on the detection of unintended remnants of foreign DNA because the mutations generated by NBT could be, from a scientific and technological viewpoint, regarded as essentially equivalent to what is caused during the natural process. If this idea is accepted, it simplifies the problem to be solved; that is, only the presence/absence of the vector sequence used in the NBT process should be examined in a host genome. Although there are several useful computer programs to compare NGS sequence reads with a genome^27^, it is cumbersome that their results are not necessarily identical to each other and some further profound bioinformatics approaches are required. For this reason, it will be formidable to establish regulations for external DNA detection based on such programs. In fact, to report sequence information for GMOs, a number of requirements describing the bioinformatics analyses are listed in the guidelines from the European Commission^17^. However, as long as the detection of a vector sequence used in the NBT process is under consideration, this complicated problem can be bypassed and one can focus on the search for identical *k*-mers between two data sets. Our simulation results indicate that the 20-mer detection analysis works effectively enough for rice (Tables 2 and 3, Fig. 2) and, as this method is fairly simple, it will probably be applicable for any other agricultural species. Another benefit of the *k*-mer detection presented in this study is that unlike genome-wide off-target mutation detection, it does not require a reference genome. Since even the high-quality rice genome is still incomplete^28^ and the other published genomes of agricultural species are much more incomplete and less accurate than that of rice, a genome-wide in-depth sequence analysis inevitably becomes insufficient to thoroughly detect all the off-target mutations. In contrast, identical *k*-mer detection between a host and vector is, as we have shown in this study, quite simple and does not require elaborate bioinformatics data processing.

To capture a small DNA fragment in a genome, the sequencing coverage should be deep enough so that any short foreign DNA will not be overlooked. Our simulation indicated that if the sequencing depth is 30× or more, the possibility that an unintended short external DNA is erroneously undetected is quite small, <10^−3^ (Table 4). Therefore, the 40× coverage proposed in the previously published guidelines for GMOs^17^ could be reasonably accepted for NBT as well. Additionally, we also have to pay attention to another problem; real sequencing reads are not evenly distributed over the genome for some reasons, such as the biases of DNA fragmentation and library construction methods, and hence, there should be regions where reads are scarce. Our mapping of the real data to the rice genome showed that deep sequencing may not completely cover the entire genome, and we cannot exclude the possibility that there is a small portion of genomic sequences that remain unexamined (Supplementary Table 5). Note that the “unsequenced” regions may be due to algorithms in the computer programs used, but we were not able to distinguish such “undetected” nucleotides from the “unsequenced” ones. As long as there is uncertainty about the completeness of DNA sequencing and stringent assessments are required, one may have to consider another technology that can be coupled with sequencing so that weaknesses of a single method will be complemented.

In addition to the computer simulation, our real data analyses of rice and bread wheat also successfully detected the presence and absence of foreign DNA inserted into the genomes (Fig. 3 and Supplementary Fig. 10). Although possible foreign DNA remnants in the bread wheat genome were previously examined by means of a standard mapping program for NGS data^31^, the *k*-mer detection described in this study is more sensitive and accurate than the previous results. However, there might be cases where such short DNA segments are essentially undetectable if actual sequencing data are much more disordered and complicated than the model cases presented in this study. Furthermore, it is not clear whether these short DNA segments have a significant biological function that has a risk of health damage. Our sequence comparison indicates that, in practice, DNA sequences of 20 nt in length can be shared between rice and a vector (Table 1), such that a universally existing short DNA will not be harmful. To avoid unrealistically stringent regulations that are technologically impossible, 30 nt or even more might be appropriate, and this criterion should be determined after due consideration. As aforementioned, it would rather be suggested that different technologies, such as Southern blot assay, be conducted in parallel and be compared with massive DNA sequencing so that the assessment becomes rigorous enough.

The minute amounts of DNA contamination during sequencing are generally overlooked but can be a problem of vital significance for some specific studies such as the detection of foreign DNA in GMOs or GEAPs (Supplementary Note 2). Any analysis that aims to search for unauthorized GMOs by high-throughput sequencing should be interpreted with caution. In our *k*-mer detection, although a significant number of false *k*-mer hits derived from the contaminated DNA in a library preparation kit were observed, these hits were diminished by an appropriate statistical test (Fig. 3). Nonetheless, we should keep in mind at all times that the contamination could lead to false positives or negatives. An alternative is that, if an unexpectedly large number of *k*-mer hits are found in a wild type sample, it is recommended to use another library preparation kit (Supplementary Fig. 8).

In the next few years NBT will become possible in a large number of species as genome editing was recently applied to a minor orphan crop^33^. Thus, it is anticipated that NBT including genome editing will be prevalent for creation of novel agricultural products from a wide variety of species. To cope with this new era of biotechnology, for example, the Ministry of Environment and some other related ministries in Japan made a statement about their policies for NBT regulations, in which the assessment of unintended remaining external DNA is required (https://www.env.go.jp/press/2_2_%20genome%20editing_En.pdf). Since NBT is expected to play a major role in not only developed countries but also developing countries^34^, safety assessment methods for GEAPs need to be cost-effective and simple without sacrificing precision. Current massive DNA sequencing methods should best fit to this type of assessment. Among high-throughput sequencing technologies, SBS has long been used for molecular biology for more than a decade. It is relatively mature enough for routine work and several investigations for practical applications of this method have been carried out for GMOs^11–16^. In this study, we showed that in addition to past conventional methods, SBS is also immediately applicable to GEAP regulation. However, we also have to note that, since the DNA sequencing technologies are still being markedly advanced, in general, any analytical methods should be reevaluated constantly. Currently, other emerging sequencing technologies, represented by single molecule real-time sequencing technologies and nanopore-based sequencing^35–37^, can produce long reads of several kilobases. While they are too error-prone at present to precisely detect small genomic structural changes, if accurate long-read sequencing methods are developed in the future, the genome-wide precise detection of DNA structural changes, such as small and large insertions, may become possible for NBT products. As long as technologies for either bioengineering or DNA sequencing are being advanced, regulations for agricultural products will have to be reconsidered and altered accordingly.

## Methods

### Data for simulation

The sequence data (accession number J01566) for the ColE1 plasmid, which was used as the cloning vector model, was downloaded from the International Nucleotide Sequence Database Collaboration^38^. The current version of the rice genome, IRGSP-1.0, was downloaded from the RAP-DB^28,39^. For the computer simulation studies, a DNA segment of a desired nucleotide length was randomly selected from the vector and was randomly inserted into the rice genome. The simulated 100-nt paired-end read data sets with an average insert length of 500 nt and a standard deviation of 50 nt were computationally generated from the rice genome and were examined using the following *k*-mer detection analysis. Since the real short reads contain a specific amount of sequencing errors, 0.3% of artificial mutations, which is modeled on the HiSeq error rate^40^, were incorporated into the simulated reads in a random manner. The examination of the accuracy of the detection method was iterated 1,000 times for each experiment.

### *K*-mer detection analysis

The *k*-mers on both strands for all the extracted reads were searched for in a vector sequence. The left ends of the identical *k-*mers on the vector were recorded and regarded as the positions of the *k*-mers. Since the vector sequence used in this study was circular, some of the first nucleotides of an appropriate length were added to the end of the vector sequence so that the *k*-mers over the boundary could be captured. For each nucleotide position in the vector, the number of identical *k*-mers and all the other *k*-mers were compared between the read sets obtained from the respective wild type genome (contrast) containing no vector insert and a DNA-segment-inserted genome. The statistical significance at the 1% level (*G* = 6.634) of the difference in these numbers was examined by a *G*-test of independence, which postulates Model II described by Sokal and Rohlf^41^, and only the cases where significantly excess *k*-mers were observed in the DNA-segment-inserted genome were adopted. In case that *k* is smaller than the segment length, if at least one of the *k*-mers in the vector region to which the segment corresponds is significant, the DNA segment insert was judged as “detected.”

### DNA alignment and assembling

To align the sequence reads to a genome, we used BWA-MEM ver. 0.7.17, NovoAlign ver. 3.09.00 (http://www.novocraft.com/) and SOAPaligner ver. 2.21 with default options^42,43^. Only the read pairs properly mapped by these programs were adopted for the comparison of the three programs, while all the mapped reads were used to examine the mapping coverage. The read depth over the genome was calculated by SAMtools ver. 1.5^44^ and the number of nucleotide positions with zero depth was counted. The *de novo* sequence assembly was carried out by SOAPdenovo2 ver. 2.04 with default options^45^.

### CRISPR/Cas9 vector construction

An all-in-one binary vector for rice, pZH_gALS-2_Cas9, which harbors *HPT*, *SpCas9* and sgRNA targeting *OsALS* (Supplementary Fig. 12), was constructed as described previously^46^. The double-stranded target sequences were created by annealing the paired single oligonucleotides and cloned into the *Bbs*I sites of pU6gRNA-oligo. OsU3-gYSA in pZH_OsU3gYSA_MMCas9 was replaced by synthetic gRNA expression constructs at the *Asc*I and *Pac*I sites^46^.

### Transformation of rice callus

We conducted the *Agrobacterium*-mediated transformation of rice (*Oryza sativa* L. cv. Nipponbare) using scutellum-derived calli, following a previously published protocol^47^. The one-month-old cultured rice calli were infected with *Agrobacterium* strain EHA105^48^. After 3 days of co-cultivation, the calli were transferred to callus-induction medium containing 50 mg/l hygromycin B and 25 mg/l meropenem (Wako Pure Chemical industries Ltd.). Hygromycin-resistant calli were selected after callus induction over 4 weeks and transferred to regeneration medium to obtain the regenerated plants.

### Experimental confirmation of the vector-free nature

Southern blot assay was performed as described previously with some minor modifications^49^. The rice genomic DNA from the wild type and T_0_ plants was isolated by the CTAB method^50^. The genomic DNA (10 µg) was digested with *Spe*I. The *HPT* gene fragment was amplified by PCR using the HPT-F01 and HPT-R01 primers (Supplementary Table 6) and was used as a probe for the Southern blot assay. To confirm the null segregate transformants in the T_1_ generation, *HPT* was amplified by PCR with the same primers described in Supplementary Table 6. The PCR experiment was performed under the following conditions: initial denaturation at 94 °C for 5 min; 30 cycles of 98 °C for 15 sec, 55 °C for 30 sec, and 68 °C for 50 sec; and final extension at 68 °C for 5 min.

### DNA sequencing and analysis

The whole-genome sequencing of rice was conducted using the Illumina HiSeq X platform to obtain 151-nt paired-end reads. The genomic DNA libraries were constructed by using the TruSeq DNA PCR-Free Library Preparation Kit (Illumina, Inc.). The total nucleotide numbers of the reads were as follows: 63,610,009,038 for wild type sample no. 1; 71,368,754,156 for wild type sample no. 2; 69,170,747,118 for the T_0_ sample; and 63,340,672,150 for the T_1_ sample. For bread wheat, the BioProject accession number PRJDB7455 was downloaded from the DDBJ Sequence Read Archive^31^. The possible adapter sequences and low-quality regions were discarded by Trimmomatic ver. 0.36 with the following options: ILLUMINACLIP:TruSeq_custom.fa:2:30:10 LEADING:10 TRAILING:10 SLIDINGWINDOW:4:20 MINLEN:20^51^, in which the TruSeq_custom.fa file contained all the adapter sequences from Illumina’s reagent kits. After trimming, the numbers of nucleotides of rice were as follows: 52,292,878,177 for wild type sample no. 1; 58,863,113,535 for wild type sample no. 2; 60,730,824,843 for the T_0_ sample; and 54,313,317,595 for the T_1_ sample. To assess the statistical significance of the wild type samples, two independent samples were compared. For the statistical test for T_0_ and T_1_, wild type sample no. 1 was used as a control. After trimming of the wheat data, the numbers of nucleotides were: 525,287,826,316 for the wild type sample; 554,268,624,561 for the T_0_ sample; and 512,633,803,717 for the T_1_ sample.

## Supplementary information

Supplementary Information.

## Data Availability

All DNA sequence data of rice analyzed in this study are available at the DDBJ Sequence Read Archive under the accession number of PRJDB8199.

## Code availability

The programs used for the *k*-mer detection analyses in this work are available at GitHub (https://github.com/taitoh1970/kmer).
