# Supplementary material for: Foreign DNA detection by high-throughput sequencing to regulate genome-edited agricultural products
Source: Sci Rep. 2020 Mar 18;10:4914. doi: 10.1038/s41598-020-61949-5 (PMC7080720; doi:10.1038/s41598-020-61949-5)
Supplement: Supplementary file 1 — Supplementary Information. [file 41598_2020_61949_MOESM1_ESM.pdf]

Foreign DNA detection by high-throughput sequencing to regulate genome-edited agricultural products (Supplementary Information)

Takeshi Itoh (ORCID: 0000-0002-6291-4233)[1,2,*], Ritsuko Onuki[1,2,6], Mai Tsuda[3], Masao Oshima[2,3], Masaki Endo (ORCID: 0000-0002-9199-181X)[2,4], Hiroaki Sakai[1,2], Tsuyoshi Tanaka[1,5], Ryo Ohsawa[3], and Yutaka Tabei[2,4]

[1]Bioinformatics Team, Advanced Analysis Center, National Agriculture and Food Research Organization, Tsukuba, Ibaraki 305-8602, Japan

[2]National Institute of Agrobiological Sciences, Tsukuba, Ibaraki 305-8602, Japan

[3]Tsukuba Plant Innovation Research Center, University of Tsukuba, Tsukuba, Ibaraki 305-8572, Japan

[4]Institute of Agrobiological Sciences, National Agriculture and Food Research Organization, Tsukuba, Ibaraki 305-8634, Japan

[5]Institute of Crop Science, National Agriculture and Food Research Organization, Tsukuba, Ibaraki 305-8518, Japan

[6]Present address: Research Institute, National Cancer Center Japan, Chuo-ku, Tokyo 104-0045, Japan.

[*]To whom correspondence may be addressed. Email: taitoh@affrc.go.jp.

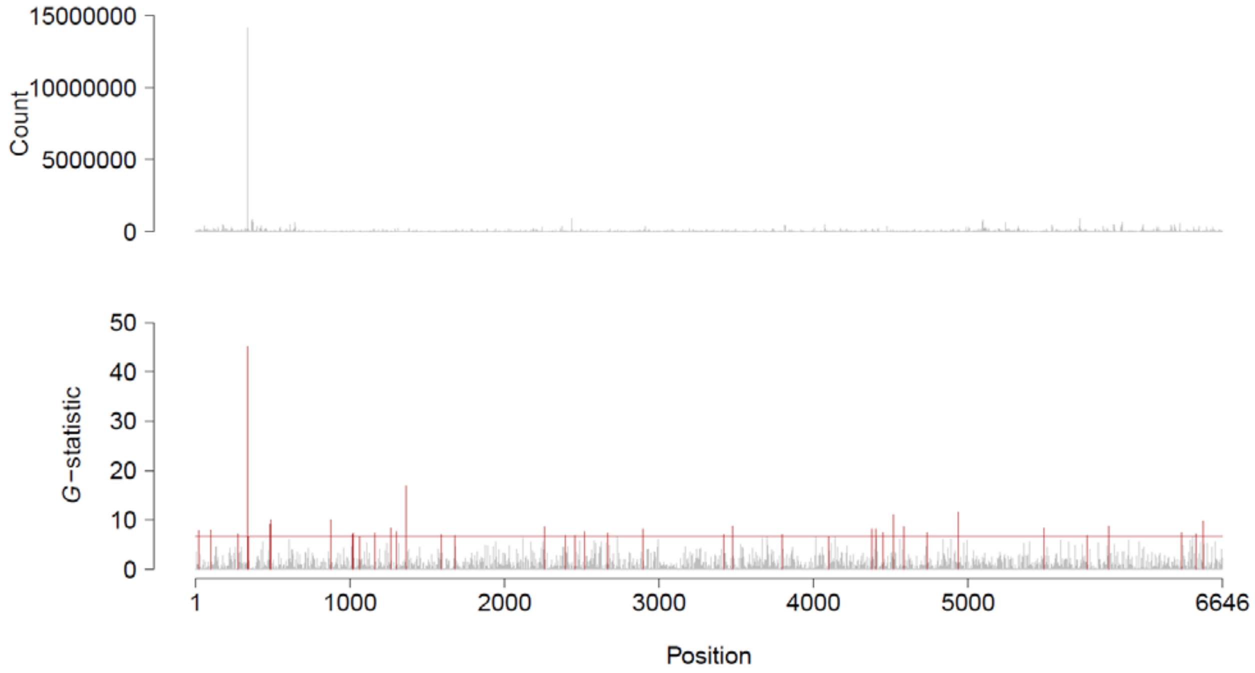

Supplementary Figure 1. A complete version of Fig. 2b.

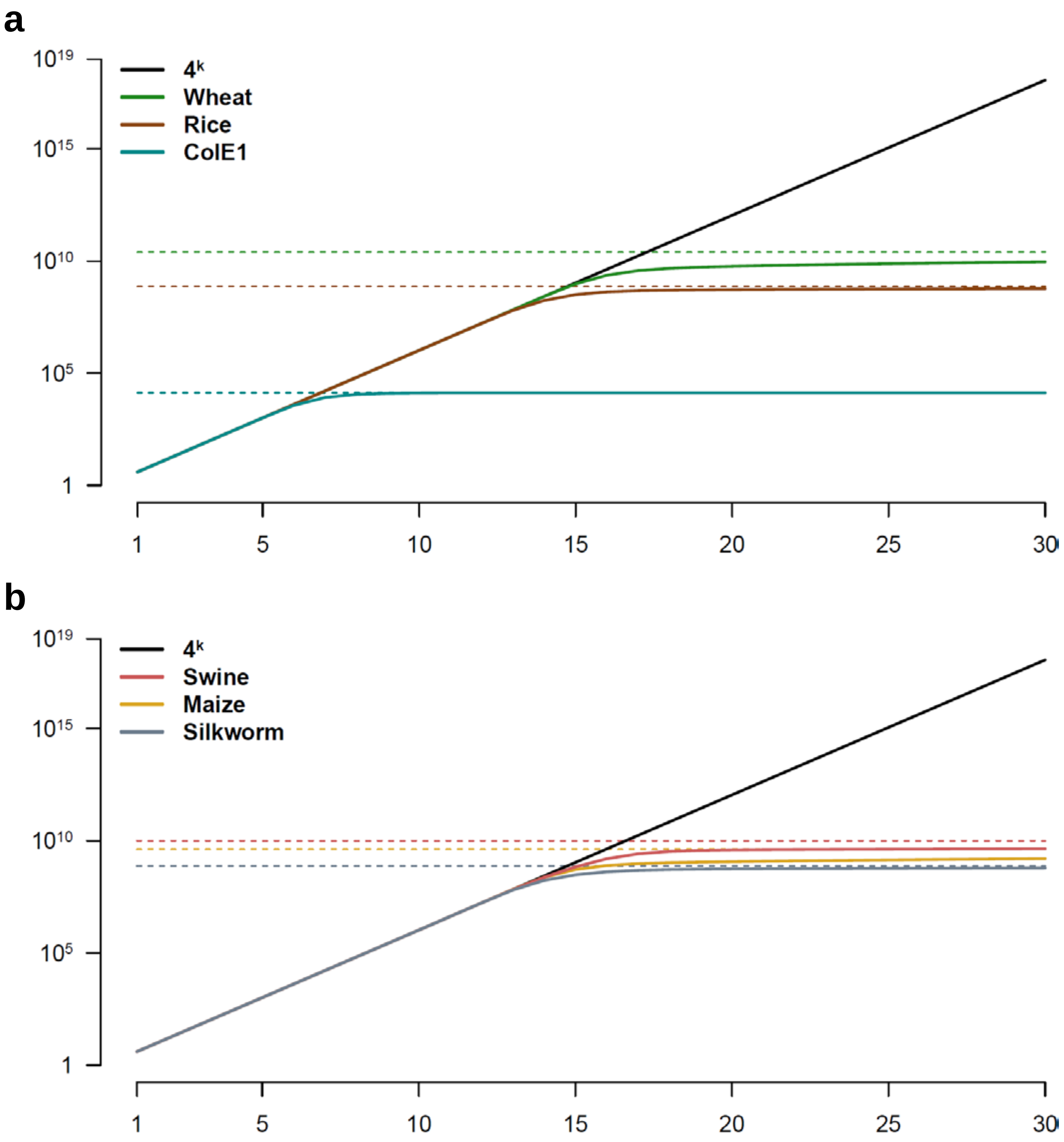

Supplementary Figure 2. The total number of *k*-mer patterns in the genomes. (**a**) Wheat, rice and ColE1. (**b**) Swine, maize and silkworm. The x-axis indicates the *k* values. The y-axis is log-scaled. The maximum numbers (4^k) are represented by a black line. The dashed lines are the theoretical upper limit of the *k*-mer numbers. The genome sequences were downloaded from the following sites: wheat (TGACv1), http://plants.ensembl.org/; swine (Sscrofa11.1), http://www.ensembl.org/; maize (Zm-B73-REFERENCE-GRAMENE-4.0), https://www.maizegdb.org/; and silkworm (as of December 12, 2017), http://sgp.dna.affrc.go.jp/. For rice and ColE1, see the Methods section.

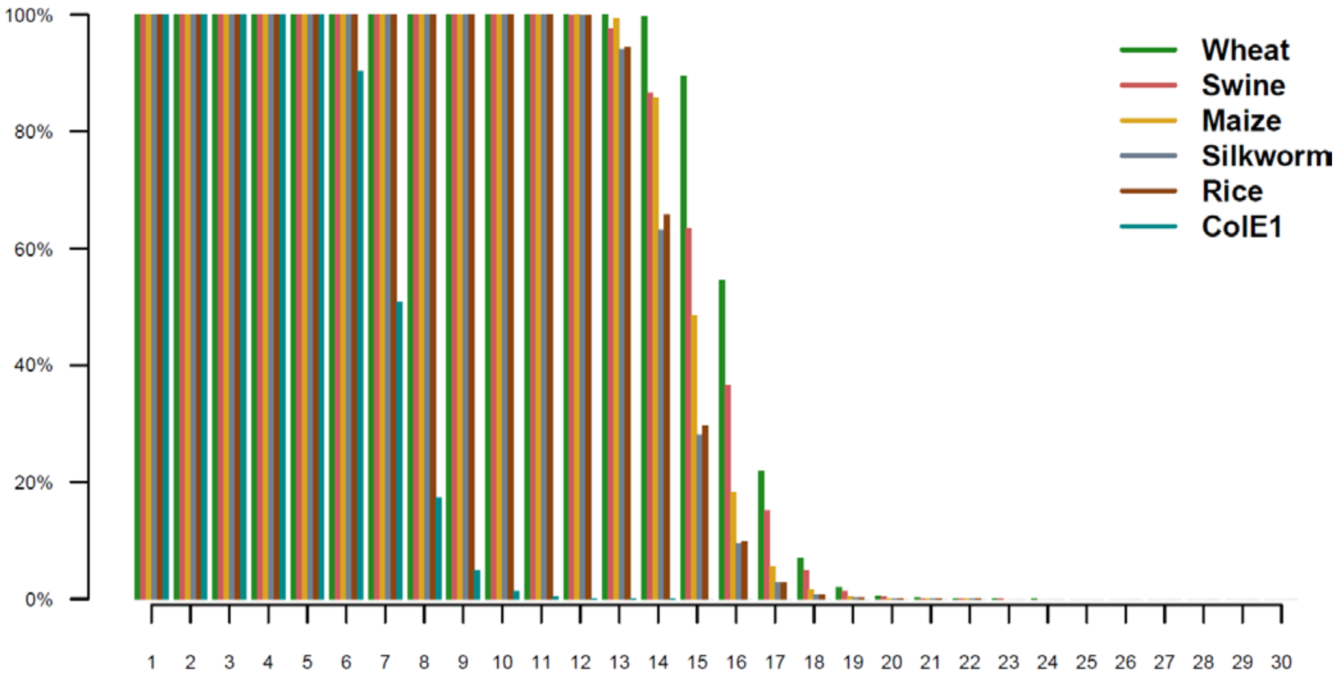

Supplementary Figure 3. The occupancy ratios of the *k*-mer patterns in genomes. The data used are the same as those in Supplementary Fig. 2.

a

b

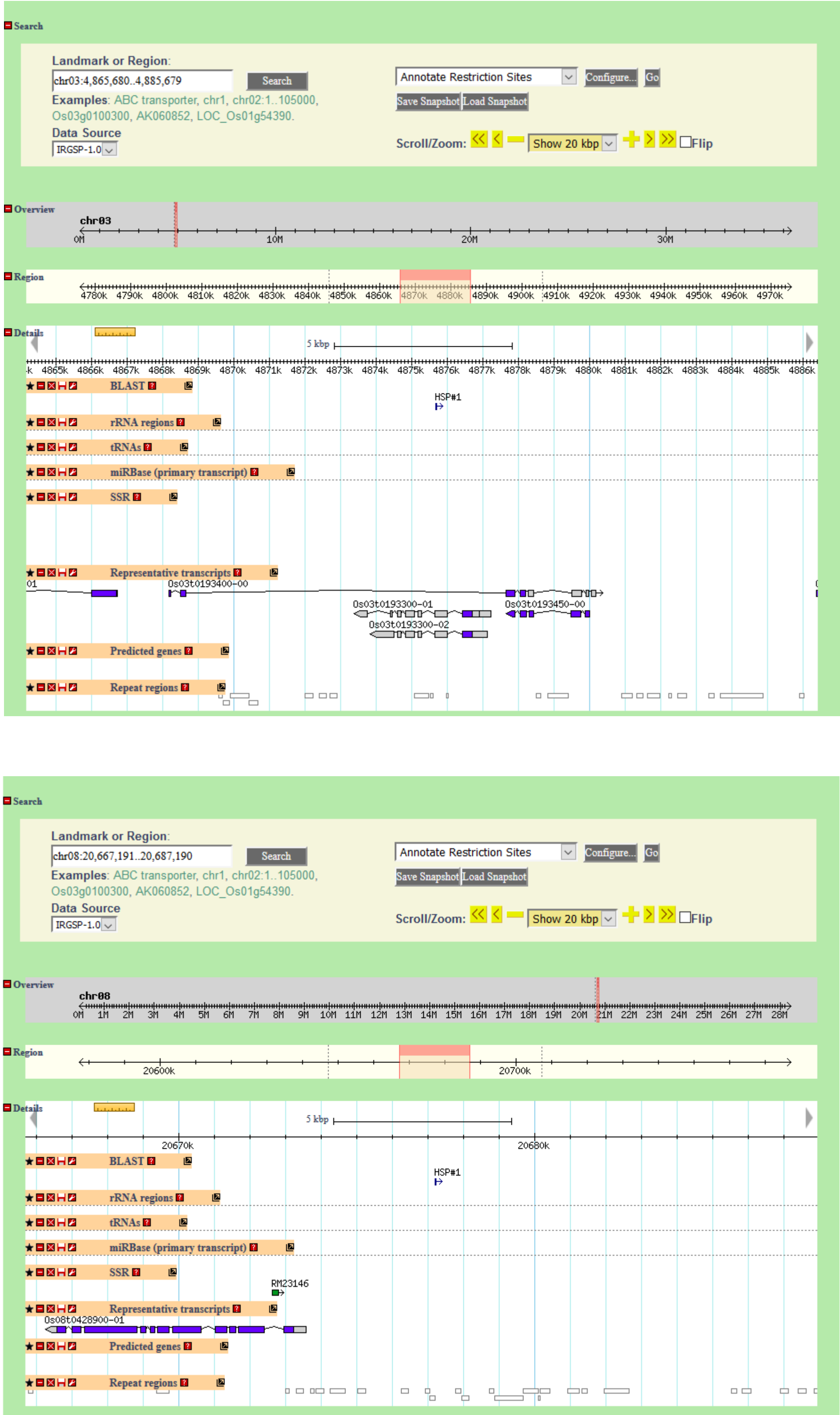

Supplementary Figure 4. Two 20-nt sequences that were identical between rice and ColE1. (**a**) 4,875,670-4,875,689 bp (ACAAGGAATTTCCTGTTCCC) on chromosome 3 and (**b**) 20,677,181-20,677,200 bp (GCATAAATAGGTTTAATTTT) on chromosome 8. These two sequences are indicated by "HSP#1" on the BLAST track. The sequences were searched for and visualized in RAP-DB (https://rapdb.dna.affrc.go.jp/).

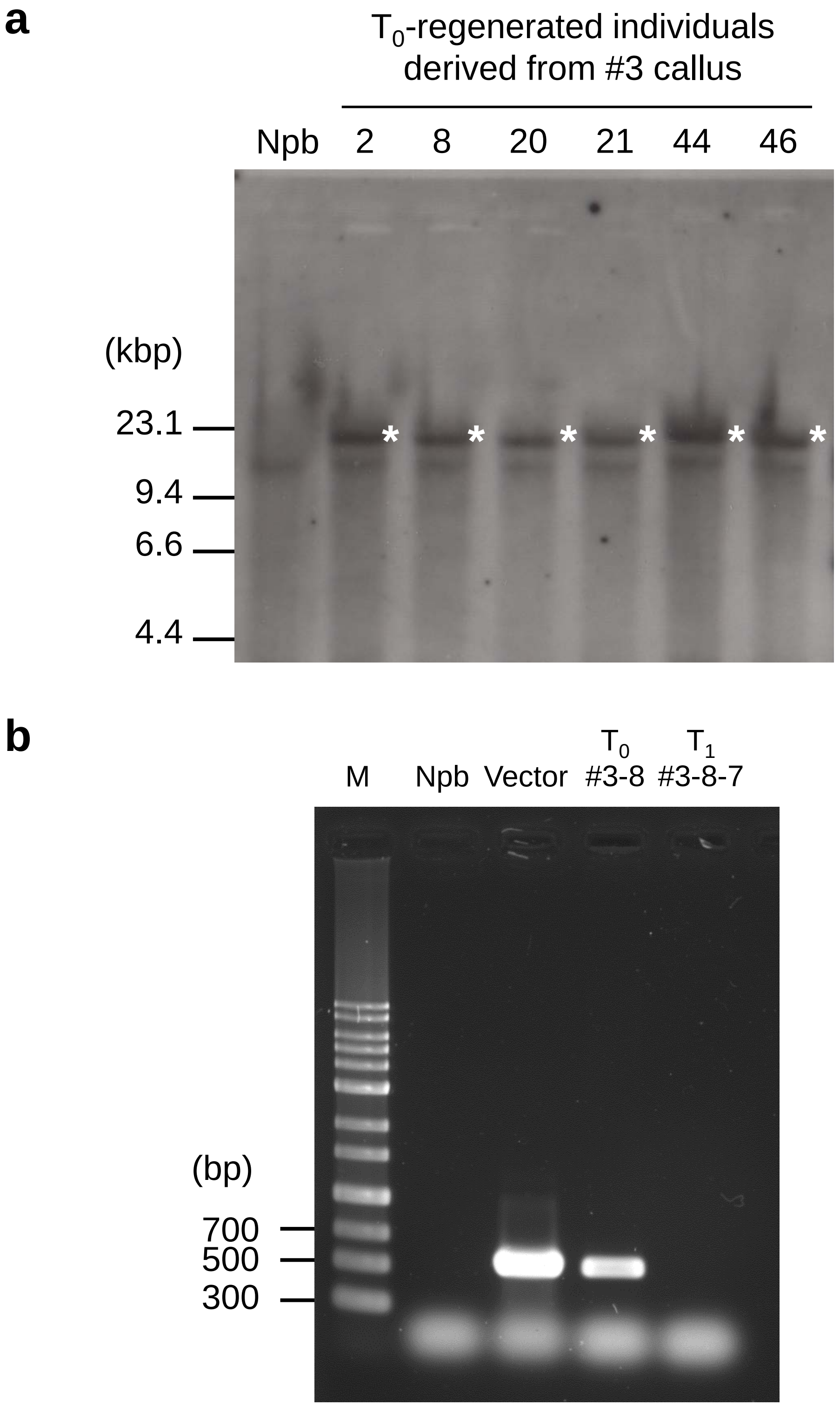

Supplementary Figure 5. Detection of null segregants. (**a**) Southern blot analysis was conducted for the T_0-regenerated individuals derived from a callus named "#3." Each lane represents a T_0-regenerated individual derived from a single callus. Nipponbare (Npb) is used as a control. Asterisks indicate the signal specific to T_0 plants. (**b**) A PCR experiment was conducted to confirm a null segregant in T_1 plants. For T_0, "8" from #3 (#3-8) was selected and #3-8-7 was used for T_1. M: 1-kbp "DNA Ladder One" marker (Nacalai Tesque, Kyoto, Japan). Neither groupings of cropped gel images nor modifications of the images were made for (**a**) and (**b**).

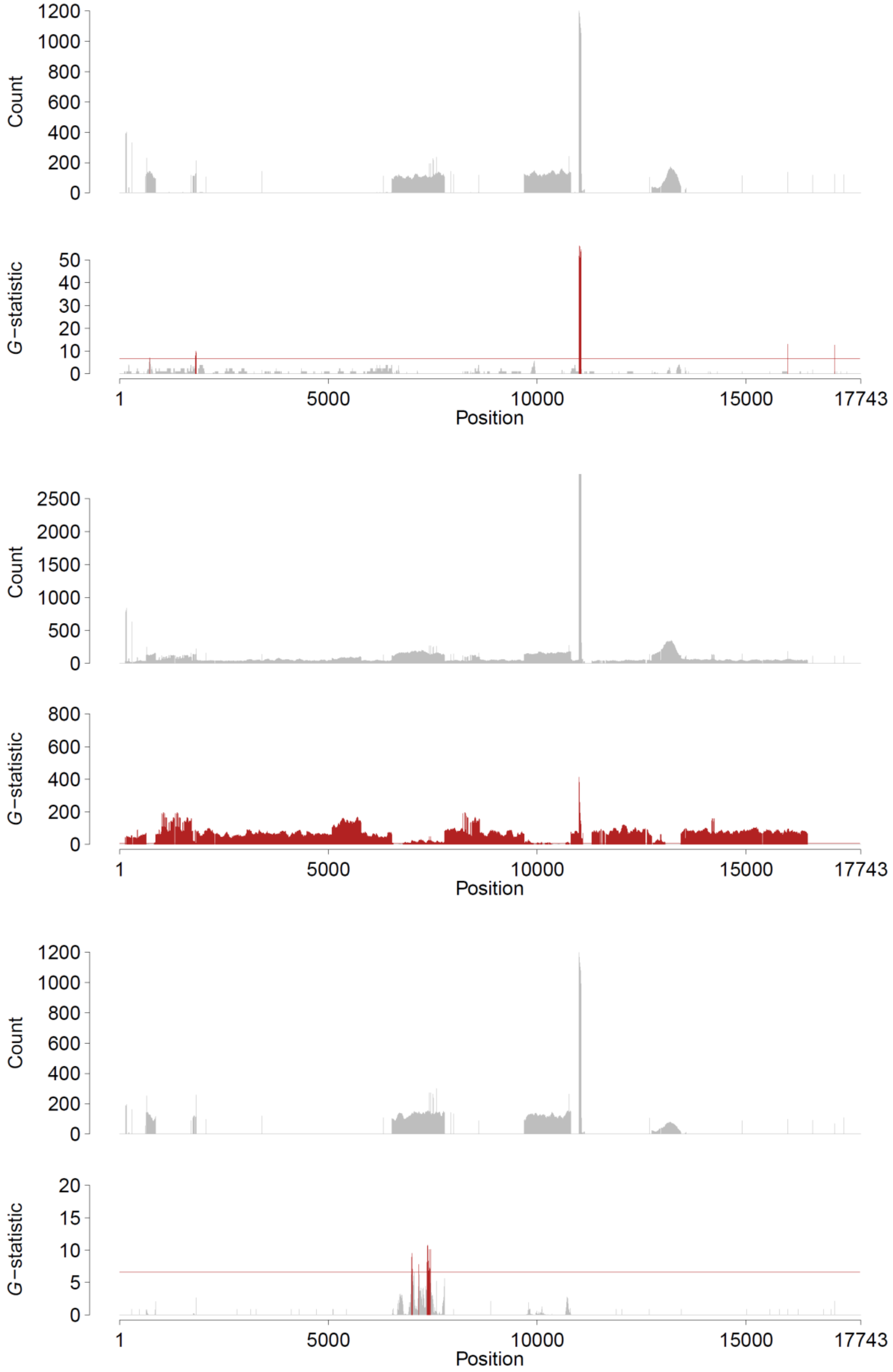

Supplementary Figure 6. A complete version of Fig. 3.

```
CLUSTAL 2.1 multiple sequence alignment


wild_type       ----GTCGTATTACGTCGCGCTCACTGGCCGTCGTTTTACAACGTCGTGACTGGGAAAAC
T1              GTGAGTCGTATTACGTCGCGCTCACTGGCCGTCGTTTTACAACGTCGTGACTGGGAAAAC
                    ********************************************************

wild_type       CCTGGCGTTACCCAACTTAATCGCCTTGCAGCACATCCCCCTTTCGCCAGCTGGCGTAAT
T1              CCTGGCGTTACCCAACTTAATCGCCTTGCAGCACATCCCCCTTTCGCCAGCTGGCGTAAT
                ************************************************************

wild_type       AGCGAAGAGGCCCGCACCGAAACGCCCTTCCCAACAGTTGCGCAGCCTGAATGGCGAATG
T1              AGCGAAGAGGCCCGCACCGAAACGCCCTTCCCAACAGTTGCGCAGCCTGAATGGCGAATG
                ************************************************************

wild_type       GGAGCGCCCTGTAGCGGCCACTCAACCCTATCTCGGTCTATTCTTTTGATTTATAAGGGA
T1              GGAGCGCCCTGTAGCGGCCACTCAACCCTATCTCGGTCTATTCTTTTGATTTATAAGGGA
                ************************************************************

wild_type       TTTTGCCGATTTCGGCCTATTGGTTAAAAAATGAGCTGATTTAACAAAAATTTAACGCGA
T1              TTTTGCCGATTTCGGCCTATTGGTTAAAAAATGAGCTGATTTAACAAAAATTTAACGCGA
                ************************************************************

wild_type       ATTTTAACAAAATATTAACGCTTACAATTTAGGTGGCACTTTTCGGGGAAATGTGCGCGG
T1              ATTTTAACAAAATATTAACGCTTACAATTTAGGTGGCACTTTTCGGGGAAATGTGCGCGG
                ************************************************************

wild_type       AACCCCTATTTGTTTATTTTTCTAAATACATTCAAATATGTATCCGCTCATGAGACAATA
T1              AACCCCTATTTGTTTATTTTTCTAAATACATTCAAATATGTATCCGCTCATGAGACAATA
                ************************************************************

wild_type       ACCCTGATAAATGCTTCAATAATATTGAAAAAGGAAGAGTATGCGTAGCCGTAATTGGAG
T1              ACCCTGATAAATGCTTCAATAATATTGAAAAAGGAAGAGTATGCGTAGCCGTAATTGGAG
                ************************************************************

wild_type       CCGTACCCTGACCGAACGTAGCGGCGGTAATGGTGCGGTGGCGGTGTTTATGGCGTGCTA
T1              CCGTACCCTGACCGAACGTAGCGGCGGTAATGGTGCGGTGGCGGTGTTTATGGCGTGCTA
                ************************************************************

wild_type       TGATTGCTTTTTTGGCGTGCAGAGCATGCCGCGTGCGAGCAAACAGCAGGCGCGTTATGC
T1              TGATTGCTTTTTTGGCGTGCAGAGCATGCCGCGTGCGAGCAAACAGCAGGCGCGTTATGC
                ************************************************************

wild_type       GGTGGGCCGTTGCCTGATGCTGTGGAGCAGCAACGATGTGACCCAGCAGGGCAGCCGTCC
T1              GGTGGGCCGTTGCCTGATGCTGTGGAGCAGCAACGATGTGACCCAGCAGGGCAGCCGTCC
                ************************************************************

wild_type       GAAAACCAAACTGAACATTATGCGTGAAGCGGTGATTGCGGAAGTGAGCACCCAGCTGTC
T1              GAAAACCAAACTGAACATTATGCGTGAAGCGGTGATTGCGGAAGTGAGCACCCAGCTGTC
                ************************************************************

wild_type       TGAAGTGGTGGGCGTGATTGAACGTCATCTGGAACCGACCCTGCTGGCCGTGCATCTGTA
T1              TGAAGTGGTGGGCGTGATTGAACGTCATCTGGAACCGACCCTGCTGGCCGTGCATCTGTA
                ************************************************************

wild_type       TGGCAGCGCGGTGGATGGCGGCCTGAAACCGCATAGCGATATTGATCTGCTGGTGNNNNN
T1              TGGCAGCGCGGTGGATGGCGGCCTGAAACCGCATAGCGATATTGATCTGCTGGTGACCNN
                *******************************************************   **

wild_type       NNNNNNNNNNNNNNNNNNNNNNNNNNNNNNNNNNNNNNNNNNNNNNNNNNNNNNNNNNNN
T1              NNNNNNNNNNNNNNNNNNNNNNNNNNNNNNNNNNNNNNNNNNNNNNNNNNNNNNNNNNNN
                ************************************************************

wild_type       GCCCGGGTGAAAGCGAAATTCTGCGTGCGGTGGAAGTGACCATCGTGGTGCATGATGACA
T1              NNNNNNNNNNNNNNNNNNNNNNNNNNNNNNNNNNNNNNNNNNNNNNNNNNNNNNNNNNNN


wild_type       TTATTCCGTGGCGTTATCCGGCGAAACGTGAGCTGCAGT---------------------
T1              NNNNNNNNNNNNNNNNNNNNNNNNNNNNNNNNNNNNNNNCGGGTGAAAGCGAAATTCTGC


wild_type       ------------------------------------------------------------
T1              GTGCGGTGGAAGTGANNNNNNNNNNNNNNNNNNNNNNNNNNNNNNNNNNNNNNNNNNNNN


wild_type       ------------------------------------------------------------
T1              NNNNNNNNNNNNNNNNNNNNNNNCCATCGTGGTGCATGATGACATTATTCCGTGGCGTTA


wild_type       -----------------------TTGGCGAATGGCAGCGTAACGATATTCTGGCCGGCAT
T1              TCCGGCGAAACGTGAGCTGCAGTTTGGCGAATGGCAGCGTAACGATATTCTGGCCGGCAT
                                       *************************************

wild_type       TTTTGAACCGGCGACCATTGATATCGATCTGGCCATTCTGCTGACCAAAGCGCGTGAACA
T1              TTTTGAACCGGCGACCATTGATATCGATCTGGCCATTCTGCTGACCAAAGCGCGTGAACA
                ************************************************************

wild_type       TAGCGTGGCGCTGGTTGGTCCGGCAGCGGAAGAACTGTTTGATCCGGTGCCGGAACAGGA
T1              TAGCGTGGCGCTGGTTGGTCCGGCAGCGGAAGAACTGTTTGATCCGGTGCCGGAACAGGA
                ************************************************************

wild_type       CCTGTTTGAAGCGCTGAACGAAACCCTGACCCTGTGGAACAGCCCGCCGGATTGGGCGGG
T1              CCTGTTTGAAGCGCTGAACGAAACCCTGACCCTGTGGAACAGCCCGCCGGATTGGGCGGG
                ************************************************************

wild_type       TGATGAACGCAACGTGGTGCTGACCCTGAGCCGTATTTGGTATAGCGCGGTGACCGGCAA
T1              TGATGAACGCAACGTGGTGCTGACCCTGAGCCGTATTTGGTATAGCGCGGTGACCGGCAA
                ************************************************************

wild_type       AATTGCGCCGAAAGATGTGGCGGCGGATTGGGCCATGGAACGTCTGCCGGCGCAGTATCA
T1              AATTGCGCCGAAAGATGTGGCGGCGGATTGGGCCATGGAACGTCTGCCGGCGCAGTATCA
                ************************************************************

wild_type       GCCGGTGATTCTGGAAGCGCGTCAGGCGTATCTGGGCCAGGAAGAAGATCGTCTGGCCAG
T1              GCCGGTGATTCTGGAAGCGCGTCAGGCGTATCTGGGCCAGGAAGAAGATCGTCTGGCCAG
                ************************************************************

wild_type       CCGTGCGGATCAGCTGGAAGAATTTGTGCATTACGTGAAAGGCGAAATTACCAAAGTGGT
T1              CCGTGCGGATCAGCTGGAAGAATTTGTGCATTACGTGAAAGGCGAAATTACCAAAGTGGT
                ************************************************************

wild_type       GGGTAAATAATAACTGTCAGACCAAGTTTACTCATATATACTTTAGATTGATTTAAAACT
T1              GGGTAAATAATAACTGTCAGACCAAGTTTACTCATATATACTTTAGATTGATTTAAAACT
                ************************************************************

wild_type       TCATTTTTAATTTAAAAGGATCTAGGTGAAGATCCTTTTTGATAATCTCATGACCAAAAT
T1              TCATTTTTAATTTAAAAGGATCTAGGTGAAGATCCTTTTTGATAATCTCATGACCAAAAT
                ************************************************************

wild_type       CCCTTAACGTGAGTTTTCGTTCCACTGAGCGTCAGACCCCGTAGAAAAGATCAAAGGATC
T1              CCCTTAACGTGAGTTTTCGTTCCACTGAGCGTCAGACCCCGTAGAAAAGATCAAAGGATC
                ************************************************************

wild_type       TTCTTGAGATCCTTTTTTTCTGCGCGTAATCTGCTGCTTGCAAACAAAAAAACCACCGCT
T1              TTCTTGAGATCCTTTTTTTCTGCGCGTAATCTGCTGCTTGCAAACAAAAAAACCACCGCT
                ************************************************************

wild_type       ACCAGCGGTGGTTTGTTTGCCGGATCAAGAGCTACCAACTCTTTTTCCGAAGGTAACTGG
T1              ACCAGCGGTGGTTTGTTTGCCGGATCAAGAGCTACCAACTCTTTTTCCGAAGGTAACTGG
                ************************************************************

wild_type       CTTCAGCAGAGCGCAGATACCAAATACTGTTCTTCTAGTGTAGCCGTAGTTAGGCCACCA
T1              CTTCAGCAGAGCGCAGATACCAAATACTGTTCTTCTAGTGTAGCCGTAGTTAGGCCACCA
                ************************************************************

wild_type       CTTCAAGAACTCTGTAGCACCGCCTACATACCTCGCTCTGCTAATCCTGTTACCAGTGGC
T1              CTTCAAGAACTCTGTAGCACCGCCTACATACCTCGCTCTGCTAATCCTGTTACCAGTGGC
                ************************************************************

wild_type       TGCTGCCAGTGGCGATAAGTCGTGTCTTACCGGGTTGGACTCAAGACGATAGTTACCGGA
T1              TGCTGCCAGTGGCGATAAGTCGTGTCTTACCGGGTTGGACTCAAGACGATAGTTACCGGA
                ************************************************************

wild_type       TAAGGCGCAGCGGTCGGGCTGAACGGGGGGTTCGTGCACACAGCCCAGCTTGGAGCGAAC
T1              TAAGGCGCAGCGGTCGGGCTGAACGGGGGGTTCGTGCACACAGCCCAGCTTGGAGCGAAC
                ************************************************************

wild_type       GACCTACACCGAACTGAGATACCTACAGCGTGAGCTATGAGAAAGCGCCACGCTTCCCGA
T1              GACCTACACCGAACTGAGATACCTACAGCGTGAGCTATGAGAAAGCGCCACGCTTCCCGA
                ************************************************************

wild_type       AGGGAGAAAGGCGGACAGGTATCCGGTAAGCGGCAGGGTCGGAACAGGAGAGCGCACGAG
T1              AGGGAGAAAGGCGGACAGGTATCCGGTAAGCGGCAGGGTCGGAACAGGAGAGCGCACGAG
                ************************************************************

wild_type       GGAGCTTCCAGGGGGAAACGCCTGGTATCTTTANTAGTCCTGTCGGGTTTCGCCACCTCT
T1              GGAGCTTCCAGGGGGAAACGCCTGGTATCTTTAT-AGTCCTGTCGGGTTTCGCCACCTCT
                *********************************  *************************

wild_type       GACTTGAGCGTCGATTTTTGTGATGCTCGTCAGGGGGGCGGAGCCTATGGAAAAACGCCA
T1              GACTTGAGCGTCGATTTTTGTGATGCTCGTCAGGGGGGCGGAGCCTATGGAAAAACGCCA
                ************************************************************

wild_type       GCAACGCGGCCTTTTTACGGTTCCTGGCCTTTTGCTGGCCTTTTGCTCATTAGGCACCCC
T1              GCAACGCGGCCTTTTTACGGTTCCTGGCCTTTTGCTGGCCTTTTGCTCATTAGGCACCCC
                ************************************************************

wild_type       AGGCTTTACCCGAACGACCGAGCGCAGCGAGTCAGTGAGCGAGGAAGCGGAGAGCGCCCA
T1              AGGCTTTACCCGAACGACCGAGCGCAGCGAGTCAGTGAGCGAGGAAGCGGAGAGCGCCCA
                ************************************************************

wild_type       ATACGCAAGGAAACAGCTATGACCATGTTAATGCAGCTGGCACGACAGGTTTCCCGACTG
T1              ATACGCAAGGAAACAGCTATGACCATGTTAATGCAGCTGGCACGACAGGTTTCCCGACTG
                ************************************************************

wild_type       GAAAGCGGGCAGTGAAGTGACGGCCCATGAGGCCAGTTAATTAANNNNNNNNNNNNNNNN
T1              GAAAGCGGGCAGTGAAGTGAAGGCCCATGAGGCCAGNNNNNNNNNNNNNNNNNNNNNNNN
                ******************** ***************        ****************

wild_type       NNNNNNNNNNNNNNNNNNNNNNNNNNNNNNNNNNNNNNNNNNNNNNNNNNNNNNNNNNNN
T1              NNNNNNNNNNNNNNNNNNNNNNNNNNNNNNNNNNNNNNNNNNNNNNNNNNNTTAATTAAG
                ***************************************************

wild_type       NNNNNNNNNNNNNNNNNNNNNNNNNNNNNNNNNNNNNNNNNNNNNNNNNNNNNNNNNNNN
T1              CGGCCGCGATATCCTGCAGA----------------------------------------


wild_type       NNNNNNNNNNNNNNNNNNNNNNNNNNNNNNNNNNNNNNNNNGTTTGGTAAGTTGCAAATC
T1              ------------------------------------------------------------


wild_type       GAAGTTTTAGATTGAGTTCTACGTCGAGCGGCCGCGATATCCTGCAGA
T1              ------------------------------------------------
```

Supplementary Figure 7. The assembled vector-like sequences were obtained from the wild type and T_1 samples in which no vector sequences were expected. The alignment was generated by Clustal X (ver. 2.1) downloaded from http://www.clustal.org/clustal2/.

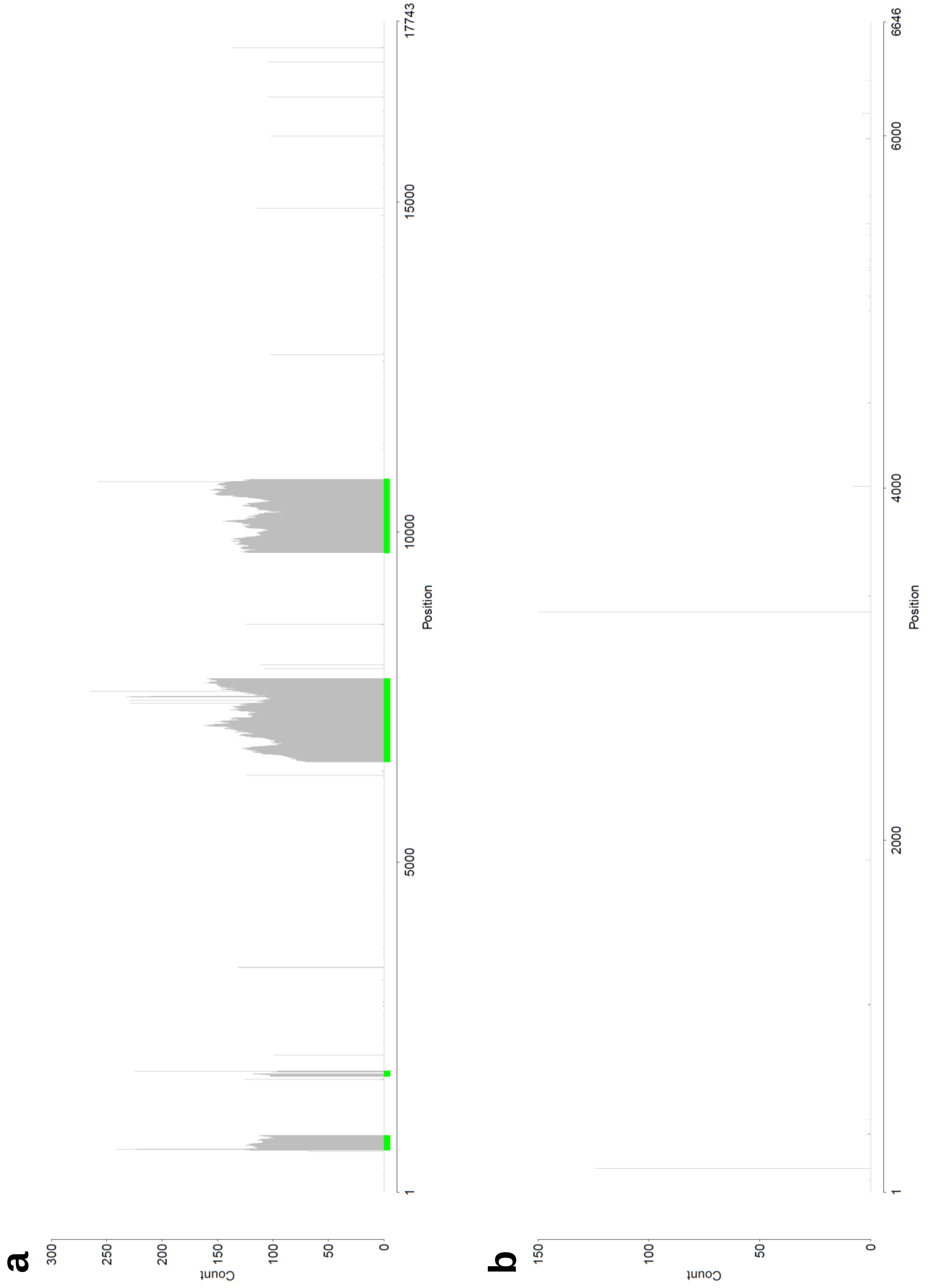

Supplementary Figure 8. Detection of identical 20-mers between the real genome and vector sequences. KAPA's library preparation kit was used. The reads were aligned to (**a**) the vector used for rice and (**b**) ColE1. Although some cisgenic regions (green boxes) had a number of hits in (**a**), the previously detected contamination (orange boxes in Fig. 3a) was not observed.

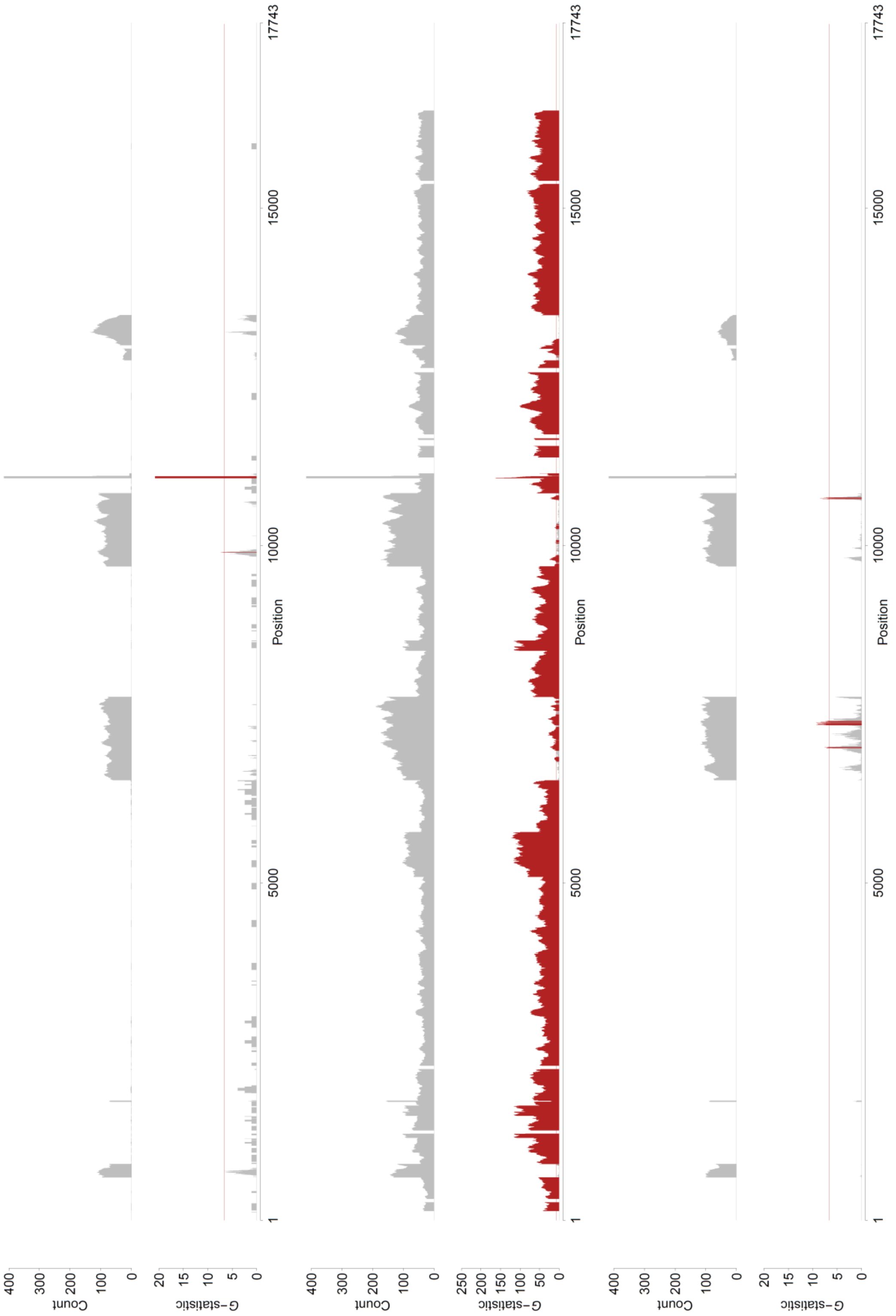

Supplementary Figure 9. Detection of identical 50-mers between the real genome and vector sequences. For details, see the legend of Fig. 3.

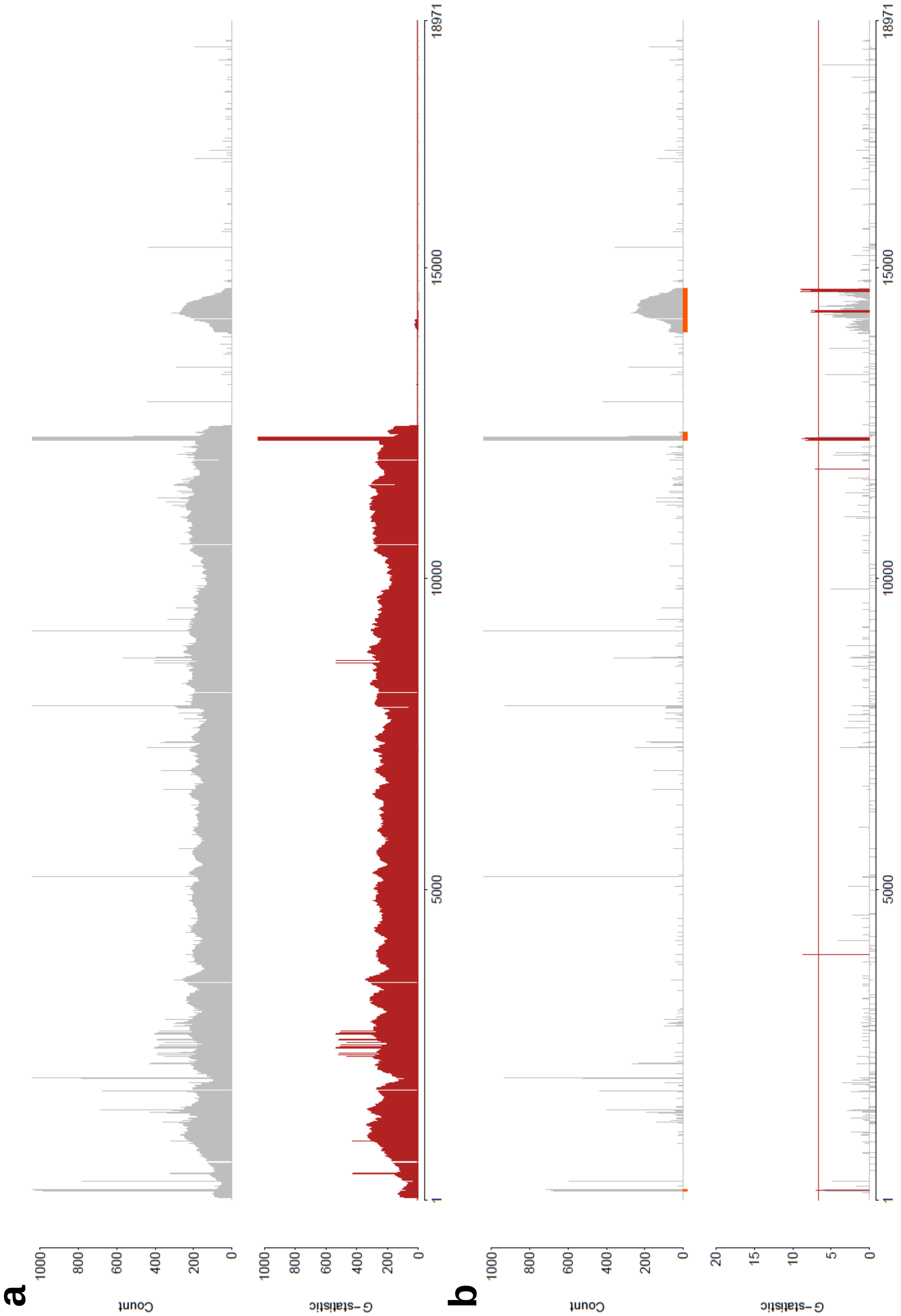

Supplementary Figure 10. Detection of identical 20-mers between the wheat genome and vector sequences. (**a**) T_0 and (**b**) T_1 were examined. The orange boxes in **b** are the regions that were previously reported as a cloning vector-like sequences (Abe, F. *et al.*, Cell Reports, 2019), which are probably due to contamination in the library preparation kit used.

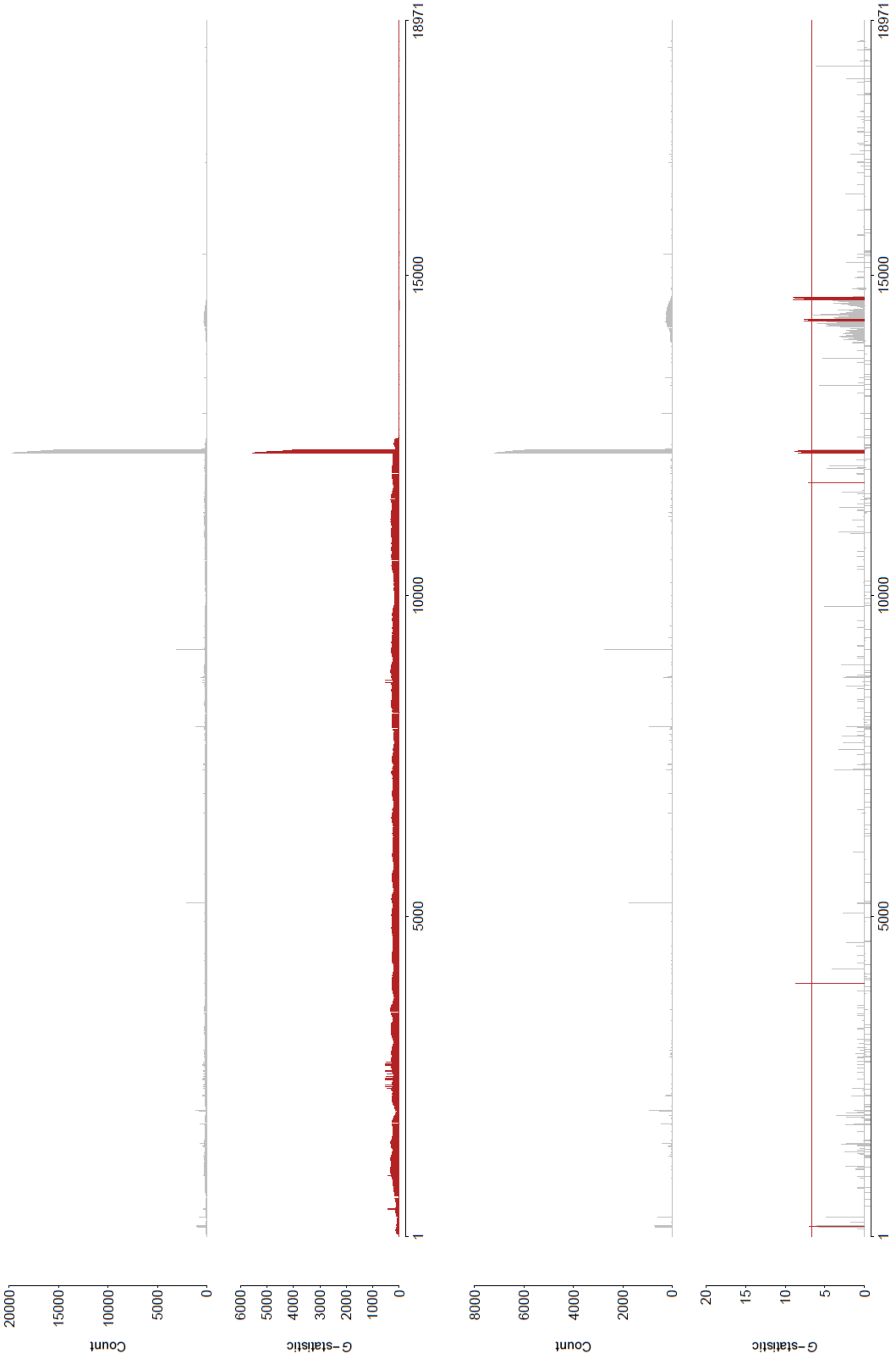

Supplementary Figure 11. A complete version of Supplementary Fig. 10.

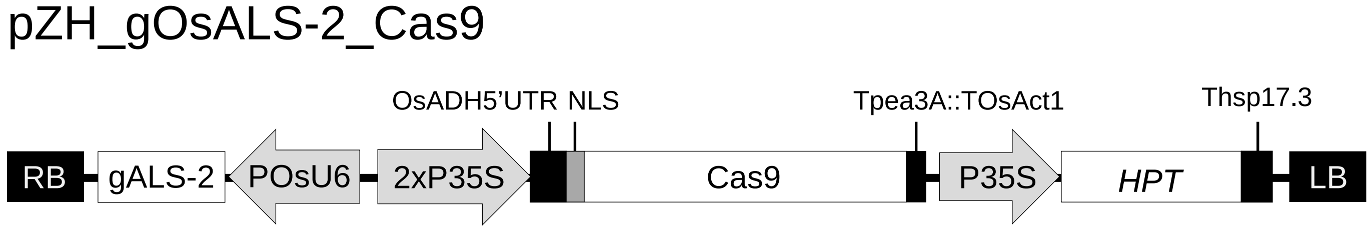

Supplementary Figure 12. Expression vector construct for sgRNA and SpCas9 in rice.

Supplementary Table 1. Number of properly mapped read pairs to the rice genome.

	Number of pairs	Mapped ratio
Total Reads (50x Coverage)	93,282,216	-
BWA-MEM	93,281,848	99.99961%
NovoAlign	88,697,864	95.08550%
SOAPaligner	92,616,102	99.28592%

Supplementary Table 2. Detection of a foreign DNA insert by *k*-mer search.

Insert length (nt)	*k*	# of positions with statistical significance*	# of erroneously detected positions
100	50	100	0
10	10	398	398

*Note: true and false hits are not distinguished.

Supplementary Table 3. Occupancy ratio of *k*-mer patterns in genomes.

k	Wheat	Maize	Rice	Swine	Silkworm	*B. subtilis*	ColE1
1	100.000%	100.000%	100.000%	100.000%	100.000%	100.000%	100.000%
2	100.000%	100.000%	100.000%	100.000%	100.000%	100.000%	100.000%
3	100.000%	100.000%	100.000%	100.000%	100.000%	100.000%	100.000%
4	100.000%	100.000%	100.000%	100.000%	100.000%	100.000%	100.000%
5	100.000%	100.000%	100.000%	100.000%	100.000%	100.000%	100.000%
6	100.000%	100.000%	100.000%	100.000%	100.000%	100.000%	90.308%
7	100.000%	100.000%	100.000%	100.000%	100.000%	100.000%	50.757%
8	100.000%	100.000%	100.000%	100.000%	100.000%	100.000%	17.400%
9	100.000%	100.000%	100.000%	100.000%	100.000%	99.819%	4.829%
10	100.000%	100.000%	100.000%	100.000%	100.000%	94.258%	1.248%
11	100.000%	100.000%	100.000%	99.999%	100.000%	67.928%	0.315%
12	100.000%	99.999%	99.920%	99.875%	99.940%	31.847%	0.079%
13	100.000%	99.375%	94.445%	97.555%	94.029%	10.590%	0.020%
14	99.670%	85.780%	65.814%	86.573%	63.220%	2.944%	0.005%
15	89.526%	48.442%	29.759%	63.401%	28.114%	0.762%	0.001%
16	54.536%	18.249%	9.853%	36.506%	9.542%	0.193%	0.000%
17	21.870%	5.521%	2.789%	15.156%	2.791%	0.048%	0.000%
18	6.901%	1.522%	0.739%	4.832%	0.756%	0.012%	0.000%
19	1.959%	0.405%	0.191%	1.343%	0.197%	0.003%	0.000%
20	0.533%	0.106%	0.049%	0.352%	0.051%	0.001%	0.000%
21	0.142%	0.028%	0.012%	0.090%	0.013%	0.000%	0.000%
22	0.038%	0.007%	0.003%	0.023%	0.003%	0.000%	0.000%
23	0.010%	0.002%	0.001%	0.006%	0.001%	0.000%	0.000%
24	0.003%	0.000%	0.000%	0.001%	0.000%	0.000%	0.000%
25	0.001%	0.000%	0.000%	0.000%	0.000%	0.000%	0.000%
26	0.000%	0.000%	0.000%	0.000%	0.000%	0.000%	0.000%
27	0.000%	0.000%	0.000%	0.000%	0.000%	0.000%	0.000%
28	0.000%	0.000%	0.000%	0.000%	0.000%	0.000%	0.000%
29	0.000%	0.000%	0.000%	0.000%	0.000%	0.000%	0.000%
30	0.000%	0.000%	0.000%	0.000%	0.000%	0.000%	0.000%

Supplementary Table 4. Average number and standard deviation of false positive hits in the detection

Coverage				
10x	20x	30x	40x	50x
0.55±0.88	0.67±0.94	0.67±0.95	0.68±0.94	0.69±0.96

Supplementary Table 5. Total number of unmapped nucleotides.

	Wild type #1	Wild type #2
BWA-MEM	59,411	58,207
NovoAlign	4,251,829	4,094,610
SOAPaligner	131,133	124,402

Supplementary Table 6. Primers used in this study for PCR of *HPT.*

Primer name	Sequence (5'–3')
HPT-F01	CAAAGATCGTTATGTTTATCGGCACTTTG
HPT-R01	GAAGAAGATGTTGGCGACCTCGTATTG

Supplementary Note 1

Throughout the real data analyses, the reads were all preprocessed by Trimmomatic so that low-quality regions would be removed prior to the *k*-mer analysis. Theoretically, this process may be omitted because as long as identical *k*-mers are our concern, all the erroneous sequence segments will simply be disregarded. We compared the preprocessed data with non-preprocessed data from the wild type sample and found that the total number of false positive sites increased from 79 to 102 in the non-preprocessed data. This is probably because sequencing errors led to a specific number of spuriously identical *k*-mers. Even though the probability that sequencing errors create these false identical hits is extremely low, the enormous amounts of reads generated by modern sequencing technologies could result in tens or hundreds of false positives. Therefore, to reduce false hits, the trimming of the low-quality regions by an appropriate program is recommended.

Supplementary Note 2

The unexpected contamination found in the wild type sequences (orange boxes in Fig. 3a) was examined as follows. The entire read sets from the wild type and T_1 samples, where no vector sequences were expected, were respectively subjected to *de novo* assembly by SOAPdenovo2. These independent assembly attempts obtained essentially the same vector-like sequence (Supplementary Fig. 7), which was partially identical to but largely different from our vector sequence. This sequence was sent to Illumina, Inc. and it was confirmed that this unexpected DNA was related to the use of DNA-binding proteins in the manufacturing of the Illumina reagent kit components (Rooz Golshani, personal communication). While this type of subtle DNA contamination would not hamper an ordinary analysis of high-throughput sequencing data, the assessment results of a generally-used vector sequence, which is a major concern for GMO and NBT regulations, should be interpreted with caution.

Since this contamination was caused by the TruSeq DNA PCR-Free Library Preparation Kit, we additionally used the KAPA Hyper Prep Kit/PCR Free and DNA sequencing was conducted by using Illumina HiSeq X to obtain 151-nt paired-end reads. After trimming by Trimmomatic, a total of 55,310,502,778 nucleotides were subjected to our *k*-mer analysis. As a result, although some false hits were observed in limited small regions. no obvious contamination signals that span several hundreds or thousands of nucleotides were observed (Supplementary Fig. 8). This indicates that if an appropriate library preparation kit was used, the contamination problem does not occur.
